# Statin Therapy and the Development of Cerebral Amyloid Angiopathy—A Rodent *in Vivo* Approach

**DOI:** 10.3390/ijms17010126

**Published:** 2016-01-19

**Authors:** Björn Reuter, Alexander Venus, Saskia Grudzenski, Patrick Heiler, Lothar Schad, Matthias Staufenbiel, Michael G. Hennerici, Marc Fatar

**Affiliations:** 1 Department of Neurology and Neurophysiology, University Freiburg, 79106 Freiburg, Germany; 2 Department of Neurology, Universitätsmedizin Mannheim, Heidelberg University, 68167 Mannheim, Germany; venus.alexander@gmx.de (A.V.); grudzenski@neuro.ma.uni-heidelberg.de (S.G.); m.g.hennerici@eurostroke.eu (M.G.H.); fatar@neuro.ma.uni-heidelberg.de (M.F.); 3 Computer Assisted Clinical Medicine, Heidelberg University, 68167 Mannheim, Germany; patrick.heiler@siemens.com (P.H.); lothar.schad@medma.uni-heidelberg.de (L.S.); 4 Nervous System Department, Novartis Institutes for Biomedical Research, 4002 Basel, Switzerland; staufenbiel.m@gmail.com

**Keywords:** CAA, transgenic mice, APP23, statins, cerebral microbleeds, amyloid

## Abstract

Background: Cerebral amyloid angiopathy (CAA) is characterized by vascular deposition of amyloid β (Aβ) with a higher incidence of cerebral microbleeds (cMBs) and spontaneous hemorrhage. Since statins are known for their benefit in vascular disease we tested for the effect on CAA. Methods: APP23-transgenic mice received atorvastatin-supplemented food starting at the age of eight months (*n* = 13), 12 months (*n* = 7), and 16 months (*n* = 6), respectively. Controls (*n* = 16) received standard food only. At 24 months of age cMBs were determined with T2*-weighted 9.4T magnetic resonance imaging and graded by size. Results: Control mice displayed an average of 35 ± 18.5 cMBs (mean ± standard deviation), compared to 29.3 ± 9.8 in mice with eight months (*p* = 0.49), 24.9 ± 21.3 with 12 months (*p* = 0.26), and 27.8 ± 15.4 with 16 months of atorvastatin treatment (*p* = 0.27). In combined analysis treated mice showed lower absolute numbers (27.4 ± 15.6, *p* = 0.16) compared to controls and also after adjustment for cMB size (*p* = 0.13). Conclusion: Despite to a non-significant trend towards fewer cMBs our results failed to provide evidence for beneficial effects of long-term atorvastatin treatment in the APP23-transgenic mouse model of CAA. A higher risk for bleeding complications was not observed.

## 1. Introduction

In the last decades the relevance of cerebral amyloid angiopathy (CAA) as a substantial human neurovascular and neurodegenerative disease was increasingly recognized. CAA is known from histopathological studies since the 1930s and was initially declared to be an orphan disease [1]. Although histopathological studies in Alzheimer’s disease (AD) research in the 1970s and 1980s demonstrated accompanying vascular amyloid β (Aβ) deposits, in particular the implementation of magnetic resonance imaging (MRI) and development of transgenic mouse models made an important contribution to our CAA knowledge and led to a burst of scientific interest and research in this field [2,3,4]. The sporadic variant of CAA is most frequent and may occur as a distinct entity or linked to AD [5]. Population-based studies indicate a prevalence of severe CAA between 7% to 24% in non-demented, and 37% to 43% in demented aged subjects [6].

CAA is characterized by the formation of oligomers and insoluble Aβ deposits in the wall of leptomeningeal and cortical small arteries and capillaries with subsequent neurovascular dysfunction [7,8]. Brain imaging reveals white matter lesions, cortical microinfarcts, superficial siderosis, and multiple cerebral microbleeds (cMBs) in a varying extent. Patients with CAA suffer a higher incidence of cognitive impairment and spontaneous lobar hemorrhage [9,10,11,12,13,14]. The spatial relationship between vascular Aβ deposition and lobar cMBs was demonstrated both by histological studies and non-invasive imaging, comparing gradient echo sequences and Pittsburgh compound B positron emission tomography (PiB-PET) [15,16,17]. Although the understanding and neuroimaging of CAA have improved remarkably, unfortunately we still face a lack of therapeutical options.

Statins are widely-used and generally well tolerated cholesterol lowering drugs. While potentially beneficial effects in AD were extensively investigated, there is only little data available on statin treatment in CAA [18]. Aside of their possible effects on Aβ production, deposition and parenchymal clearance, statins are frequently used to improve vascular dysfunction [19]. Depending on their precise mechanism of action (e.g., reduction of Aβ production *vs.* increase in clearance from parenchyma and brain interstitial fluid into the blood vessels) statins might have different effects on CAA and cMBs. To address this question, we investigated the long-term effects of atorvastatin administration in the APP23 transgenic mouse model by use of magnetic resonance imaging (MRI) and Prussian blue (PB)/Thioflavin S staining. The APP23 transgenic mouse model was originally designed as a model of AD, but also showed a relevant vascular Aβ deposition with subsequent morphological changes known from humans suffering CAA, *i.e.*, cMBs and rarely macrohemorrhage [20]. Selected outcome parameters were the amount of cMBs measured by MRI and the histologically-assessed vascular Aβ burden.

## 2. Results

### 2.1. Influence of Statin Therapy on Mortality

Treatment with atorvastatin had no significant influence on the mortality rate with 12/29 controls dying within the study period (41%) compared to 4/17 mice treated for 16 months (24%, *p* = 0.21), 4/11 mice treated for 12 months (36%, *p* = 0.78), and 5/11 mice treated for eight months (46%, *p* = 0.82). In pooled analysis 13/39 treated mice died within the study period (33%, *p* = 0.50). This left 17 controls, 13 mice treated for 16 months, seven mice treated for 12 months, and six mice treated for eight months for MRI and histological evaluation.

### 2.2. Cerebral Microbleeds

**Figure 1:**
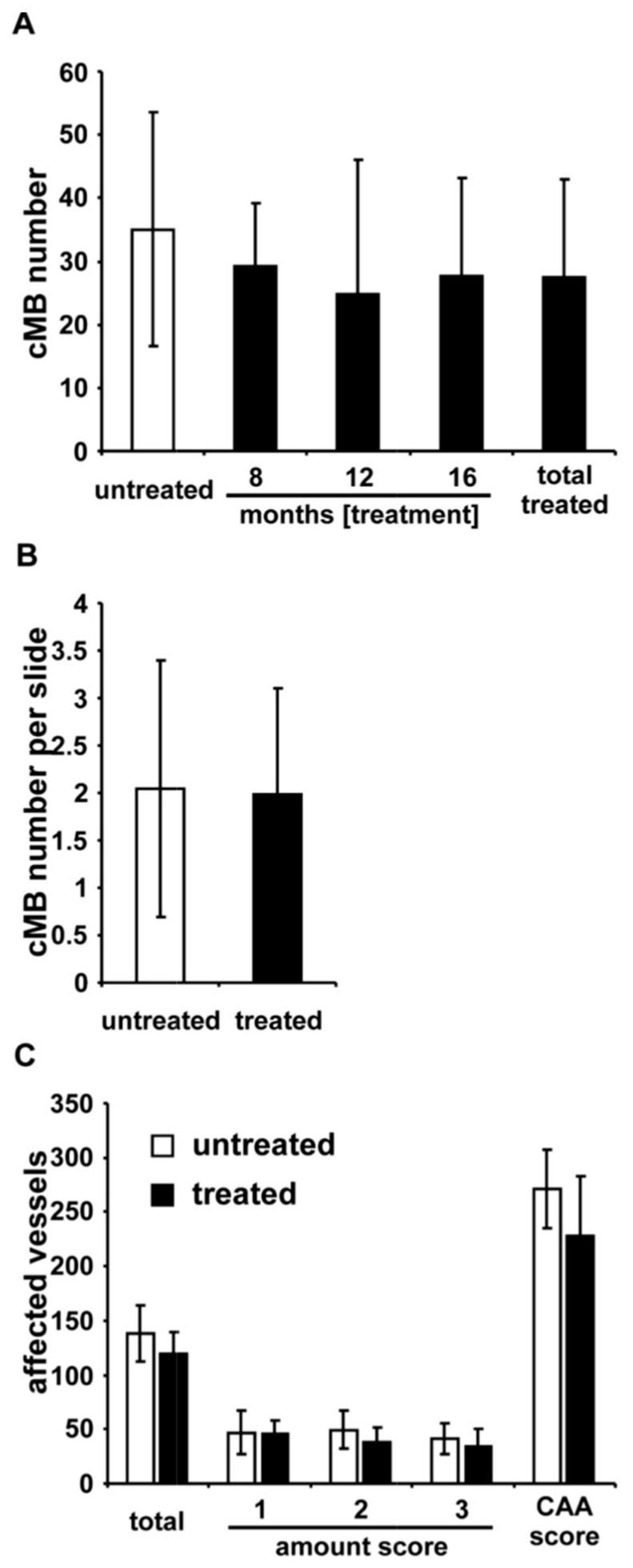
(**A**) Number of cerebral microbleeds (cMBs) in MRI and APP23-tg mice treated with atorvastatin for eight months (*n* = 6), 12 months (*n* = 7), and 16 months (*n* = 13), respectively. Compared to controls (*n* = 17) and in pooled analysis no significant differences between the groups were observed; (**B**) Histologically-assessed numbers of cMBs per slide in untreated mice (*n* = 6) did not differ significantly from those in mice treated for 16 months with atorvastatin (*n* = 7); (**C**) In these mice total numbers of thioflavin S positive vessels, graded by severity of amyloid β (Aβ) deposits, and after calculation of a cerebral amyloid angiopathy (CAA) severity score showed no significant difference between the groups.

In MRI analysis numbers of cMBs did not significantly differ between the control mice and atorvastatin treated mice, independent from treatment duration. Numbers of cMBs were 35 ± 18.5 (mean ± SD) for control mice (*n* = 16) compared to 29.3 ± 9.8 after eight months (*n* = 6, *p* = 0.49), 24.9 ± 21.3 after 12 months (*n* = 7, *p* = 0.26), and 27.8 ± 15.4 after 16 months of atorvastatin treatment (*n* = 13, *p* = 0.27; Figure 1A and Table 1).

After adjustment for size the amount of cMBs remained nonsignificant. In a secondary pooled analysis, control mice were compared to all atorvastatin-treated mice independent of the treatment duration. Numbers of cMBs were not significantly different between the groups (*p* = 0.16), as well as after adjustment for size (*p* = 0.13).

A subgroup of six randomly selected control mice and seven mice treated with atorvastatin for 16 months were analyzed histologically to validate the data derived by MRI measurements. The average number of cMBs per analyzed section was *n* = 2 ± 1.1 in treated mice and *n* = 2 ± 1.4 in the untreated control group (Figure 1B). Data were compared to MRI findings by using Pearson’s correlation analysis, which demonstrated a good correlation between both methods and, thus, the validity of MRI derived data (*r* = 0.81; *p* = 0.001) (Figure S1). Additionally, the cMB size score obtained by MRI correlated significantly with histological findings (*r =* 0.81; *p* = 0.001).

### 2.3. Vascular Amyloid β (Aβ) Deposition

The mean number of Aβ-affected vessels per mouse in APP23-tg control mice was equal with 138 ± 26 (*n* = 6) compared to 120 ± 20 in mice treated with atorvastatin for 16 months (*n* = 7, *p* = 0.18; Figure 1C). Controls had a mean CAA-Score of *n* = 271 ± 36 compared to 228 ± 55 in atorvastatin treated mice (*p* = 0.13).

**Table 1:** Average numbers of cMBs in treated and untreated APP23-tg mice in total and categorized by size and analyzed via magnetic resonance imaging (MRI).

Group	Mice (*n*)	Total cMBs	*p*-Value	cMBs ≤ 100 µm	*p*-Value	cMBs 150–200 µm	*p*-Value	cMBs > 200 µm	*p*-Value
Control group	16	35 ± 18.5	-	17.2 ± 9.7	-	12.8 ± 6.9	-	5.1 ± 5.4	-
8 months of treatment	6	29.3 ± 9.8	*p* = 0.49	16.3 ± 6.4	*p* = 0.84	9.8 ± 4.0	*p* = 0.34	3.0 ± 2.1	*p* = 0.38
12 months of treatment	7	24.9 ± 21.3	*p* = 0.26	12.9 ± 9.6	*p* = 0.33	9.3 ± 10.3	*p* = 0.34	2.7 ± 2.1	*p* = 0.28
16 months of treatment	13	27.8 ± 15.4	*p* = 0.27	13.7 ± 9.4	*p* = 0.34	10.2 ± 4.7	*p* = 0.26	3.9 ± 2.8	*p* = 0.5
pooled treatment group	26	27.4 ± 15.6	*p* = 0.16	14.1 ± 8.6	*p* = 0.29	9.9 ± 6.3	*p* = 0.17	3.4 ± 2.5	*p* = 0.26

The pooled treatment group combines all animals without consideration of treatment duration. Significance was calculated compared to the untreated control group. Data is presented as mean ± standard deviation (SD). cMBs: cerebral microbleeds.

## 3. Discussion

We present the first experimental study in a transgenic mouse model of CAA which aims to evaluate the effect of long term statin treatment on CAA and cMBs in view of such therapy in humans. CMBs are frequently discovered in T2* weighted gradient echo imaging and susceptibility weighted imaging. A lobar distribution pattern suggests CAA as their underlying vasculopathy. Recently, cMBs were classified useful to evaluate both the progression of CAA and the impact of causal therapies [21]. Statins stabilize the integrity and function of the neurovascular unit by up-regulation of the endothelial nitric oxide synthase pathway, reduction of oxidative stress, and anti-inflammatory properties [22,23,24]. Vascular experimental and clinical studies with special focus on Aβ previously demonstrated down-regulation of E-selectin, CC chemokine ligand-2, interleukin-6, matrix metalloproteinases, and isoprenylation [25,26,27,28,29,30,31]. In addition to chronic CAA-associated inflammatory stress of the vascular system, anti-inflammation under statin therapy might also be a very timely issue in CAA in light of the recent increasing number of reports describing cases of CAA-related inflammation (CAA-ri) [32,33,34]. In a transgenic mouse model of AD simvastatin administration at high doses restored cerebrovascular reactivity, measured by acetylcholine- and calcitonin gene-related peptide-induced vasodilatation, and reduced oxidative stress [35].

Although these very complementary preceding experimental findings support the idea of an improved neurovascular function, our study failed to provide evidence for a positive effect of long term atorvastatin treatment on the progression of cMBs and vascular amyloid deposits in CAA. This observation was irrespective of the duration of atorvastatin treatment, despite a positive trend in a combined analysis of all treatment groups. Nevertheless, harmful side effects under therapy were not observed either. Statins have mild anticoagulatory properties, which are suspected to be favorable in secondary ischemic stroke prevention, but also potentially harmful for those at increased risk for intracerebral hemorrhage [36,37]. Indicators for a good safety profile are that in our study we noted equal mortality rates regardless of treatment and treatment duration. As a limitation of this finding we are unable to provide data on lethal spontaneous lobar hemorrhage in treated and untreated mice, since a histological evaluation was not performed for animals dying spontaneously during the study period. Moreover, numbers of cMBs were not elevated under statin treatment. Human observational studies reported that statin intake was independently associated with a higher incidence of cMBs predominantly in lobar location, which is not supported by our rodent approach [38,39]. Finally, atorvastatin treatment did not increase vascular Aβ deposits, which is a potential side effect as a consequence of an enhanced parenchymal amyloid plaque reduction and drainage of Aβ oligomers with the brain interstitial fluid to the vessel wall [40]. A possible reason for our missing evidence for cMB reduction and thus limitation of our study might be a previously underestimated variability of cMBs in the APP23-tg mouse model. Observed numbers of cMBs at 24 months of age ranged from 7 to 82. Regarding the existing literature this was not anticipated when we planned the study. In a retrospective power analysis based on the observed mean number and standard deviation of cMBs in 24 month old APP23-tg mice, the minimum estimated sample size would be 51 mice per group (treated *vs.* untreated) in a one-sided test and under the assumption of a medium effect size of 0.5.

With our results we hope to encourage further experimental and clinical studies in their efforts to investigate statins as safe and potentially effective drugs to lower the progression of CAA.

## 4. Materials and Methods

### 4.1. Animals

Our study was approved by the Ethical Committee for Laboratory Animal Experiments at the regional council in Karlsruhe, Germany (file number 35-9185.81/G-9/10, date of approval 06 April 2010). All procedures were in accordance with institutional animal protection guidelines. Heterozygote B6,D2-TgN(Thy-APP_SWE_)-23-tg mice (APP23-tg) were provided by Matthias Staufenbiel (Novartis Institutes for BioMedical Research, Novartis Pharma AG, Basel, Switzerland) and backcrossed twice with C57BL/6 mice (Janvier, Saint Berthevin Cedex, France). The mice were kept under a 12/12 h light/dark cycle with standard food and water *ad libidum* [41]. The animal health status was monitored three times per week and the spontaneous mortality rate over the study period of 24 months was within the expected range. Grouping was performed at random and all surviving mice were taken into our analysis.

### 4.2. Statin Treatment

**Figure 2:**
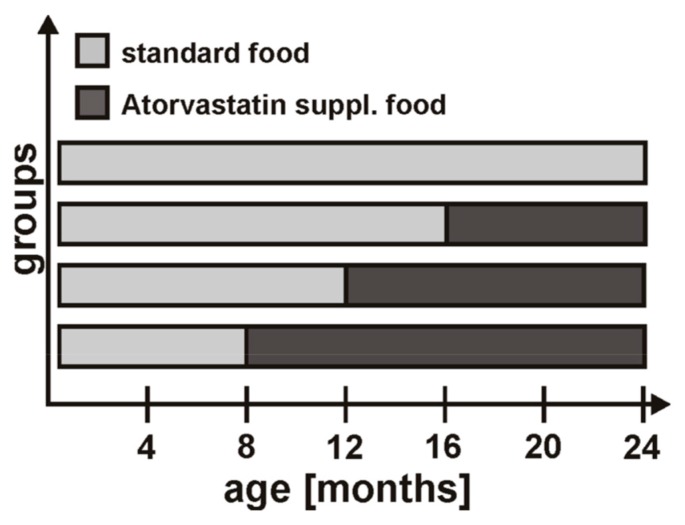
Time schedule of the study. APP23-tg mice received either standard food over the whole study period (controls, *n* = 17 with brain imaging and histology) or atorvastatin-supplemented food starting at the age of eight months (*n* = 13), 12 months (*n* = 7), and 16 months (*n* = 6), respectively.

The treatment group received standard food supplemented with 60 mg/kg atorvastatin (Sniff, Soest, Germany; Sortis^®^, Pfitzer, NY, USA) beginning at the age of eight (*n* = 17), 12 (*n* = 11), and 16 (*n* = 11) months, respectively. This corresponds to a dosage of 10 mg/kg body weight per day based on an average food consumption of 5 g/day for a 25 g mouse. APP23-tg control mice (*n* = 29) received standard food only (Figure 2). The food supplying investigator was independent from the following analyses, which were performed blinded to treatment and treatment duration. Numbers and size of cMBs were determined with magnetic resonance imaging (MRI) at the age of 24 months (fixed time endpoint).

### 4.3. Magnetic Resonance Imaging (MRI) Protocol and Analysis

*In vivo* imaging was performed on a 9.4 T Biospec 94/20 USR small animal system (Bruker, Ettlingen, Germany) equipped with 740 mT/m gradients and a 1H surface cryogenic probe [41]. Animals were anesthetised with 1.5%–2% isoflurane and positioned into the magnet with a laser-controlled system for the animal cradles. Respiratory frequency and body temperature were continuously monitored. Body temperature was maintained with a water heating pad.

Iron as a content of hemosiderin deposits leads to a decrease in the transverse relaxation time of water protons diffusing close to the cells. This results in turn in signal loss in T2* weighted gradient echo images [42,43]. Visualization was realized with a 2D-FLASH sequence and the following sequence parameters: TR/TE = 600/8 ms, flip angle = 60°, FOV = 14 × 11 mm^2^, matrix size = 256 × 200, 18 slices, three averages. The total acquisition time was 6 min.

**Figure 3:**
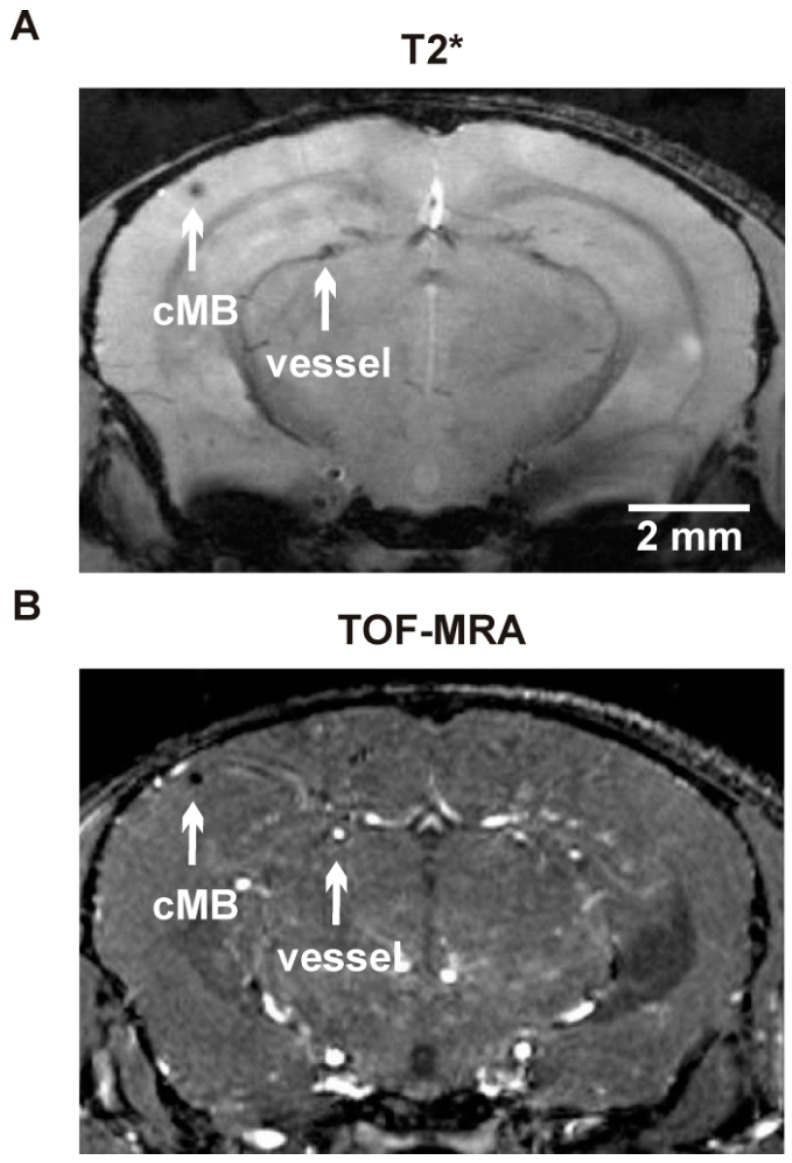
T2* weighted and time of flight magnetic resonance angiography (TOF-MRA) image of brain vessels and vessel associated cMBs in an APP23-tg mouse obtained by 9.4 T animal scanner. (**A**) Representative T2* weighted image with two hypointense areas (arrows); and (**B**) brepresentative TOF-MRA image of corresponding brain section from the same animal with a hypointense signal representing a cMB and a hyperintense signal representing a brain vessel.

**Figure 4:**
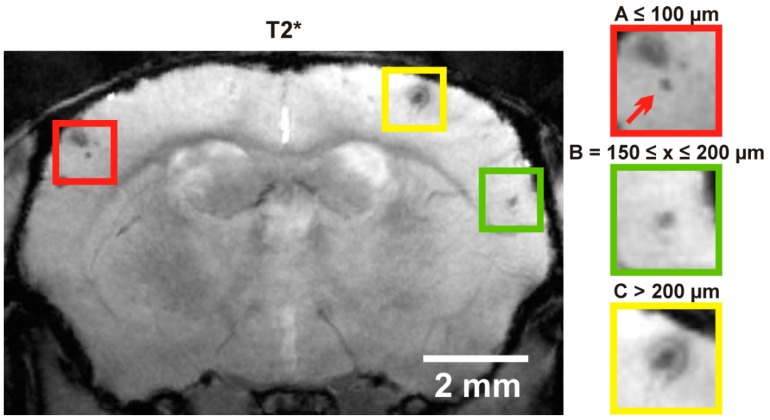
T2* weighted image of cMBs in an APP23-tg mouse obtained with 9.4 T animal scanner. Representative T2* weighted image of an APP23-tg mouse shows multiple cMBs which were graded by size (**left** image). Representative graded cMBs are shown belonging to group A ≤ 100 µm (red arrow), B = 150 ≤ x ≤ 200 µm and C > 200 µm (**right** images).

To distinguish cMBs from vessels a 3D flow-compensated gradient echo time of flight MR angiography (TOF-MRA) was performed using the following parameters: repetition time/echo time = 22/3.9 ms, flip angle = 40°; field of view = 16 × 16 × 16 mm^3^, two averages, matrix size = 256 × 256 × 128, total acquisition time = 15 min 46 s. Angiograms obtained by generating maximum intensity projections (MIPs) and analysis of amount and size of cMBs was done using ImageJ software (National Institutes of Health, Bethesda, MD, USA). Hypointense regions in T2* weighted images considered to be cMBs were verified by comparison to TOF-MRA raw data to distinguish vessel related flow void (Figure 3). CMBs were graded depending on size (Size A: cMB ≤ 100 µm, Size B: 150 ≤ cMB ≤ 200 µm, Size C: cMB > 200 µm; Figure 4).

### 4.4. Histology

Animals were sacrificed 1–3 days after MRI under deep Isoflurane anaesthesia by transcardial perfusion with 4% acid free formalin (Roth, Karlsruhe, Germany). Afterwards the brains were incubated over night in 4% acid free formalin at 4 °C, cut into blocs with thickness of 2 mm each, dehydrated in ethanol and xylol and embedded in paraffin. For histochemical analysis 4 µm sections were dewaxed in xylene and rehydrated in alcohol and distilled water. For fluorescence staining the sections were then incubated with 1% thioflavin S (Sigma-Aldrich, St. Louis, MO, USA) and 70% ethanol for five minutes respectively, as described elsewhere [44]. After washing the slides twice in 1-fold PBS (phosphate buffered saline, pH 7.4) and once in 1-fold PBS (pH 7.4)/0.3% triton for 5 min, vessel labelling was done by incubating the tissue sections with STL (biotinylated Solanum Tuberosum (Potato) Lectin, Vector Laboratories, Burlingame, CA, USA) at a ratio of 1:200 in 1-fold PBS/10% goat serum (pH 7.4) overnight at 4 °C [45]. After three additional washing steps in 1-fold PBS (pH 7.4) for 10 min each, sections were incubated with Streptavidin DyLight 594 (Vector Laboratories, Burlingame, CA, USA) and DAPI (4′,6-Diamidin-2-Phenylindol) (KPL, Gaithersburg, MD, USA) at a ratio of 1:500 for 1 h at room temperature in darkness, washed four times for 10 min each in 1-fold PBS (pH 7.4) and mounted in Mowiol (Roth, Karlsruhe, Deutschland, Germany).

**Figure 5:**
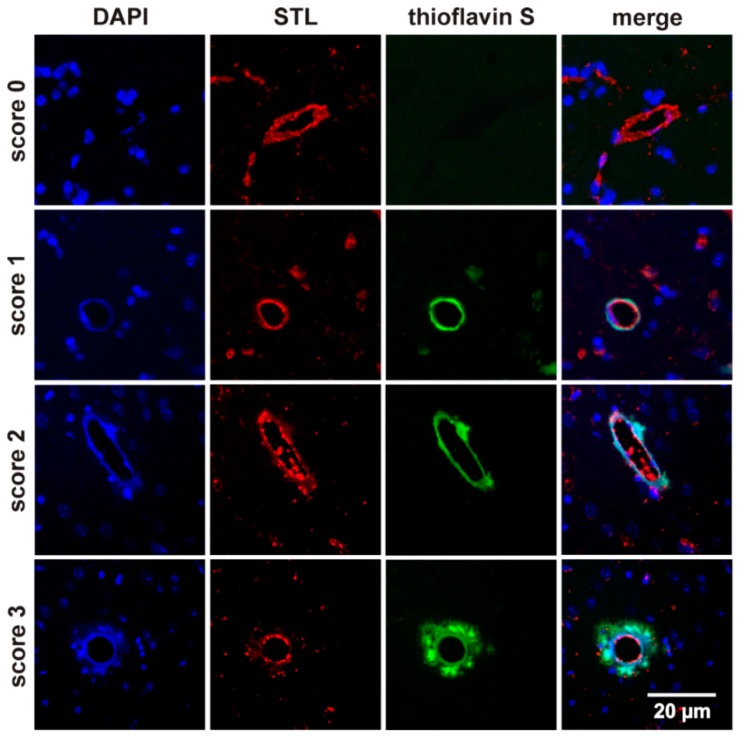
Fluorescence analysis of affected brain vessels demonstrating Aβ burden. Representative images of Aβ affected thioflavin S stained vessels in APP-tg mice. Vessels were graded into score 1, 2 or 3 depending on degree of Aβ burden. Blue = DAPI (4′,6-Diamidin-2-Phenylindol); red = STL (biotinylated Solanum Tuberosum (Potato) Lectin); green = thioflavin S.

Ten anatomically well-defined sections with two respective regions of interest were selected for histological quantification of vascular amyloid deposition. In a first step all Aβ affected vessels were quantified as described elsewhere [20]. In a second step each vessel was categorized according to the severity of Aβ deposition. Grade 1 was classified as a thin ring within the vessel wall, grade 2 as grade 1 plus additional deposits in the neuropil, and grade 3 as a ”bulky” ring of Aβ extending into the surrounding brain parenchyma. A histological “CAA Score” was calculated by multiplying numbers of affected vessels and the severity of Aβ-deposition (CAA Score = grade 1 (*n*) + grade 2 (*n*) multiplied by 2 + grade 3 (*n*) multiplied by 3) (Figure 5).

A randomly selected subgroup of 24 months old APP23-tg mice (*n* = 7 mice with 16 months of Atorvastatin therapy and *n* = 6 control mice) was used to validate MRI derived analysis of cMBs. Therefore Prussian blue (PB) stained slices (with a median of 26 slices per mouse depending on brain volume) of similar brain regions for all mice were analyzed. Numbers of hemosiderin depositions were quantified and the results were compared to the corresponding MRI data. PB staining was performed using the Accustain^®^ Iron Stain Kit (Sigma-Aldrich, St. Louis, MO, USA) according to the manufacturer’s guidelines. Nuclei were counterstained using nuclear fast red 0.1% (Merck, Darmstadt, Germany) for 10 min. Following dehydration steps in alcohol and xylol the sections were preserved in mounting medium (Eukitt, O. Kindler, Freiburg, Germany).

Bright field and fluorescence analysis were done using a Leica DM 4500 B fluorescence microscope (Leica, Wetzlar, Germany). Pictures were taken using Leica IM50 Image Manager Software (Leica, Cambridge, UK).

### 4.5. Statistical Analysis

Statistical analysis was performed with a standard software package (SPSS 19/22 for windows XP, SPPS Inc., Chicago, IL, USA). All parametric values are expressed as mean ± standard deviation (SD). Differences between the groups regarding mortality, number of cMBs, cMB size, and CAA score of vascular Aβ deposition were calculated by a two-sided independent samples *t*-test with a Levene-test for equality of variances. Findings from MRI were validated by comparing them to histological data using Pearson’s correlation. Retrospective sample size calculation with the occurrence of cMBs as a primary outcome parameter was done with G*Power (University of Düsseldorf, Germany). A *p*-value ≤0.05 was considered significant.

## 5. Conclusions

In conclusion, we investigated long term atorvastatin treatment with 10 mg/kg bodyweight per day in the APP23-tg mouse model of CAA. Outcome parameters were cMBs and vascular Aβ deposits, measured by MRI and histology. The whole brain cMB load was quickly and reliably detected *in vivo* with a 9.4 T small animal MR scanner by using a combination of T2* weighted gradient echo images and TOF-MRA raw images. MRI data was compared to histology in a subgroup of randomly selected mice and showed a good correlation. While we did not observe an increase in cMBs and histological CAA grading and thus conclude that statin therapy is safe in CAA, we failed to provide evidence for beneficial effects of atorvastatin on the progression of CAA despite a positive trend. A potential reason might be an unexpected high variance of cMBs in this mouse model at 24 months of age. Our results encourage further experimental and clinical studies to investigate statins as potential drugs to lower the progression of CAA and cMBs.

## Supplementary Materials

Supplementary materials can be found at http://www.mdpi.com/1422-0067/17/1/126/s1.

## Abbreviations

Aβ: amyloid β; AD: Alzheimer’s disease; APP23-tg: B6,D2-TgN(Thy-APP_SWE_)-23 transgenic; CAA: Cerebral amyloid angiopathy; cMB: Cerebral microbleed; DAPI: 4′,6-Diamidin-2-Phenylindol; MRI: Magnetic resonance imaging; PB: Prussian blue; PBS: Phosphate buffered saline; STL: biotinylated Solanum Tuberosum (Potato) Lectin; TOF-MRA: time of flight magnetic resonance angiography.
