# Supplementary material for: Statin Therapy and the Development of Cerebral Amyloid Angiopathy—A Rodent in Vivo Approach
Source: Int J Mol Sci. 2016 Jan 19;17(1):126. doi: 10.3390/ijms17010126 (PMC4730367; doi:10.3390/ijms17010126)
Supplement: Supplementary file 1 [file ijms-17-00126-s001.pdf]

Statin Therapy and the Development of Cerebral Amyloid Angiopathy—A Rodent *in Vivo* Approach

Björn Reuter, Alexander Venus, Saskia Grudzenski, Patrick Heiler, Lothar Schad, Matthias Staufenbiel, Michael G. Hennerici and Marc Fatar

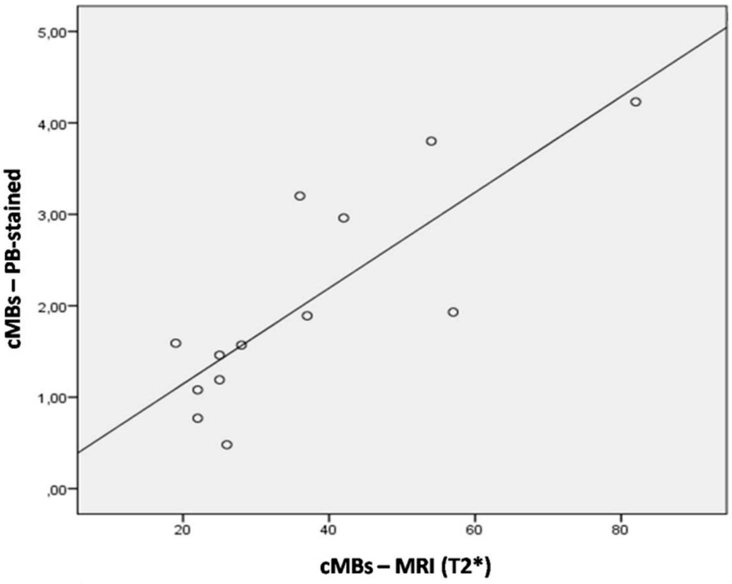

Figure S1. Correlation between histological analysis and MRI analysis of cerebral microbleeds (cMBs) in APP23 mice (n = 13). Histology: Prussian Blue—stained mice (average number of cMBs per analyzed sections). MRI: total number of analyzed cMBs in T2*-weighed MR-images.
